# 568. Using Machine Learning to Predict Place-Based Risks for *Staphylococcus aureus* Infections in Children

**DOI:** 10.1093/ofid/ofac492.621

**Published:** 2022-12-15

**Authors:** Xiting Lin, Fatima Ali, Michael R Edelson, Robert C Jerris, Traci Leong, Peter T Baltrus, Lilly Immergluck

**Affiliations:** Morehouse School of Medicine, Atlanta, Georgia; Morehouse School of Medicine, Atlanta, Georgia; InterDev, Atlanta, Georgia; Emory University School of Medicine/Children's Healthcare of Atlanta, Atlanta, Georgia; Emory University, atlanta, Georgia; Morehouse School of Medicine, Atlanta, Georgia; Morehouse School of Medicine, Atlanta, Georgia

## Abstract

**Background:**

Community-onset *Staphylococcus aureus* (CO-*S. aureus*) pediatric infections, methicillin-resistant *S. aureus* (MRSA) and methicillin-susceptible *S. aureus* (MSSA) continue to contribute to the burden of infections seen in the ambulatory setting in the US. Individual risk factors have been identified, but place-based factors and specific geographic locality have not been well-studied. The purpose of this study is to predict place-based factors that contribute to the spread of CO-*S. aureus* in a major urban area using maximum entropy (MaxEnt), a machine learning technique.

**Methods:**

Electronic medical records from two pediatric hospitals (2002 to 2016) were retrospectively reviewed. Inclusion criteria: a confirmed *S. aureus* infection within 48 hours of hospital admission (CO-*S. aureus*), < 19 years old, and a geo-referenced address within Atlanta’s metropolitan statistical area (MSA). Fourteen place-based factors, at the US Census block group level, were included in the MaxEnt models: < 18 years old, Caucasian, African American, ethnicity, poverty, education attainment, crowding, daycare, kindergarten enrollment, distance to K-12 school, distance to a children’s hospital, distance to a daycare center, and population density. A total of four models (CO-MRSA early, CO-MSSA early, CO-MRSA later, and CO-MSSA later) were run using the MaxEnt software. For each model, 75% and 25% of data was randomly assigned to training and testing groups, respectively. Models were assessed by jack-knife tests.

**Results:**

16,124 records met eligibility criteria for MaxEnt models. The training Area Under the Curve (AUC) ranged from 0.771 to 0.837 and the test AUC ranged from 0.769 to 0.804. Population density had the highest contribution in predicting CO-MRSA and CO-MSSA locations, which was confirmed by jack-knife tests.

**Conclusion:**

By applying MaxEnt to pediatric CO-*S. aureus* infections in the Atlanta MSA, it was found that higher risks of CO-*S. aureus* infections may exist in more densely populated areas. MaxEnt can be utilized to identify potential future areas of CO-MRSA and CO-MSSA infections based on estimated or predicted changes to the place-based factors used to build these models.

**Disclosures:**

**Lilly Immergluck, MD, MS**, GSK: Clinical Trial- PI|Merck: Vaccine Trial Site- serve as PI|Moderna: Board Member|Novavax: Part of CoVID-19 Phase 3 Trial through US Covid Prevention Network.

